# 1-(6-Chloro-2-methyl-4-phenyl­quinolin-3-yl)-3-(3-methoxy­phen­yl)prop-2-en-1-one

**DOI:** 10.1107/S1600536809052179

**Published:** 2009-12-09

**Authors:** Wan-Sin Loh, Hoong-Kun Fun, S. Sarveswari, V. Vijayakumar, B. Palakshi Reddy

**Affiliations:** a X-ray Crystallography Unit, School of Physics, Universiti Sains Malaysia, 11800 USM, Penang, Malaysia; b Organic Chemistry Division, School of Advanced Sciences, VIT University, Vellore-632 014, India

## Abstract

In the title compound, C_26_H_20_ClNO_2_, the quinoline ring system is approximately planar with a maximum deviation of 0.028 (2) Å and forms a dihedral angle of 73.84 (5)° with the phenyl ring. Two neighbouring mol­ecules are arranged into a centrosymmetric dimer through a pair of inter­molecular C—H⋯Cl inter­actions. A pair of inter­molecular C—H⋯O hydrogen bonds link two methoxy­phenyl groups into another centrosymmetric dimer, generating an *R*_2_^2^(8) ring motif. The structure is further stabilized by C—H⋯π inter­actions.

## Related literature

For background to and the biological activity of quinolines, see: Michael (1997 ▶); Markees *et al.* (1970 ▶); Kalluraya & Sreenivasa (1998 ▶); Chen *et al.* (2001 ▶). For the biological activity of chalcones, see: Dimmock *et al.* (1999 ▶); Zi & Simoneau (2005 ▶). For related structures, see: Fun *et al.* (2009 ▶); Loh *et al.* (2009 ▶). For hydrogen-bond motifs, see: Bernstein *et al.* (1995 ▶). For bond-length data, see: Allen *et al.* (1987 ▶). For the stability of the temperature controller used for the data collection, see: Cosier & Glazer (1986 ▶).
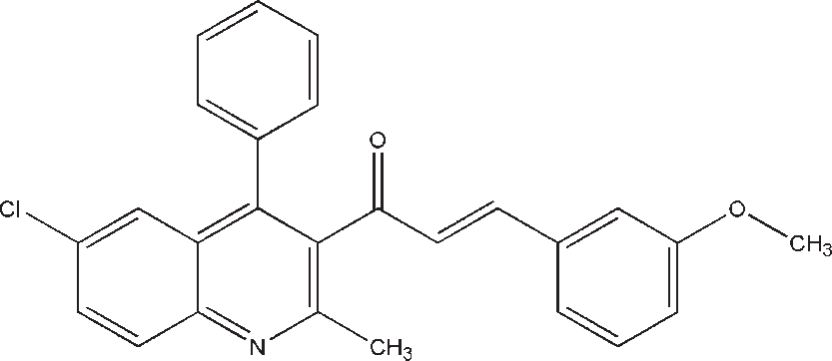


## Experimental

### 

#### Crystal data

C_26_H_20_ClNO_2_
*M* *_r_* = 413.88
Monoclinic, 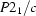

*a* = 15.6338 (2) Å
*b* = 14.0408 (2) Å
*c* = 10.0321 (1) Å
β = 108.462 (1)°
*V* = 2088.82 (5) Å^3^
*Z* = 4
Mo *K*α radiation
μ = 0.21 mm^−1^
*T* = 100 K
0.33 × 0.25 × 0.17 mm

#### Data collection

Bruker SMART APEXII CCD area-detector diffractometer
Absorption correction: multi-scan (*SADABS*; Bruker, 2005 ▶) *T*_min_ = 0.936, *T*_max_ = 0.967
51550 measured reflections
6303 independent reflections
5132 reflections with *I* > 2σ(*I*)
*R*_int_ = 0.042

#### Refinement

*R*[*F* ^2^ > 2σ(*F* ^2^)] = 0.042
*wR*(*F* ^2^) = 0.112
*S* = 1.05
6303 reflections
273 parameters
H-atom parameters constrained
Δρ_max_ = 0.38 e Å^−3^
Δρ_min_ = −0.41 e Å^−3^

### 

Data collection: *APEX2* (Bruker, 2005 ▶); cell refinement: *SAINT* (Bruker, 2005 ▶); data reduction: *SAINT*; program(s) used to solve structure: *SHELXTL* (Sheldrick, 2008 ▶); program(s) used to refine structure: *SHELXTL*; molecular graphics: *SHELXTL* software used to prepare material for publication: *SHELXTL* and *PLATON* (Spek, 2009 ▶).

## Supplementary Material

Crystal structure: contains datablocks global, I. DOI: 10.1107/S1600536809052179/is2501sup1.cif

Structure factors: contains datablocks I. DOI: 10.1107/S1600536809052179/is2501Isup2.hkl

Additional supplementary materials:  crystallographic information; 3D view; checkCIF report

## Figures and Tables

**Table 1:** Hydrogen-bond geometry (Å, °)

*D*—H⋯*A*	*D*—H	H⋯*A*	*D*⋯*A*	*D*—H⋯*A*
C16—H16*A*⋯O2^i^	0.93	2.40	3.3005 (15)	163
C18—H18*A*⋯Cl1^ii^	0.93	2.78	3.6948 (13)	169
C26—H26*B*⋯O1^iii^	0.96	2.54	3.4329 (17)	155
C26—H26*C*⋯*Cg*1^iv^	0.96	2.88	3.8412 (15)	177
C17—H17*A*⋯*Cg*2^v^	0.93	2.97	3.7592 (14)	144

Symmetry codes: (i) 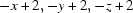; (ii) 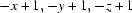; (iii) 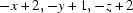; (iv) 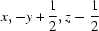; (v) 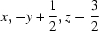. *Cg*1 and *Cg*2 are the centroids of the C13–C18 and C19–C24 rings, respectively.

**Fig. 1.**
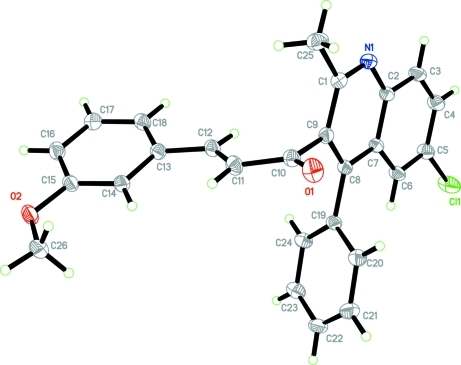
The molecular structure of the title compound, showing 50% probability displacement ellipsoids and the atom-numbering scheme.

**Fig. 2.**
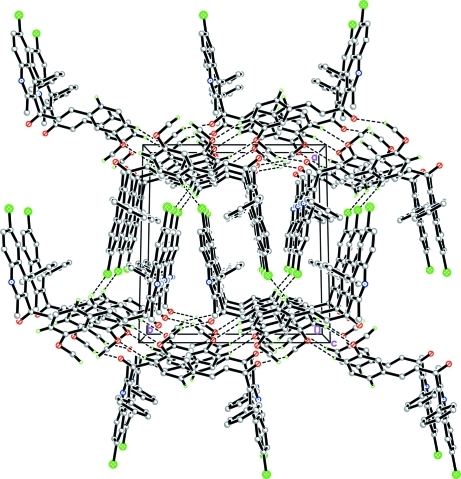
The crystal packing of the title compound, viewed along the c axis. H atoms not involved in the intermolecular interactions (dashed lines) have been omitted for clarity.

## supplementary crystallographic information

### Comment

The quinolines and their derivatives are very important compounds because of their wide occurrence in natural products (Michael, 1997) and biologically active compounds (Markees *et al.*, 1970). A large variety of quinolines have interesting physiological activities and found attractive applications as pharmaceuticals, agrochemicals and as synthetic building blocks (Kalluraya & Sreenivasa, 1998; Chen *et al.*, 2001). The chalcones are open-chain flavonoids, possessing a variety of biological activities, including antioxidant, anti-inflammation, antimicrobial, antiprotozoal, antiulcer, as well as other properties (Dimmock *et al.*, 1999). Importantly, chalcones have shown several anticancer activities as inhibitors of cancer cell proliferation, carcinogenesis and metastasis (Zi & Simoneau, 2005).

In the title compound (Fig. 1), the quinoline ring system (C1–C9/N1) is approximately planar with a maximum deviation of 0.036 (1) Å at atom C11. This mean plane of quinoline ring forms a dihedral angle of 73.84 (5)° with the phenyl ring (C19–C24). Bond lengths (Allen *et al.*, 1987) and angles are within the normal range and are comparable to closely related structures (Fun *et al.*, 2009; Loh *et al.*, 2009).

In the crystal packing (Fig. 2), two molecules are arranged into a large dimer by a pair of intermolecular C18—H18A···Cl1 interactions. A pair of intermolecular C16—H16A···O2 hydrogen bonds link two methoxyphenyl groups of the neighbouring molecules into another set of dimer, generating an *R*_2_^2^(8) ring motif (Bernstein *et al.*, 1995). The crystal structure is further stabilized by C—H···π interactions (Table 1), involving C13–C18 (centroid *Cg*1) and C19–C24 (centroid *Cg*2) rings.

### Experimental

To the solution of 3-acetyl-6-chloro-2-methyl-4-phenylquinoline (2.95 g, 0.01 *M*), 3-methoxybenzaldehyde (1.36 g, 0.01 *M*) and a catalytic amount of KOH in distilled ethanol was added and stirred for about 12 h. The resulting mixture was concentrated to remove the ethanol and then poured onto ice and neutralized with diluted acetic acid. The resultant solid was filtered, dried and purified by column chromatography using 1:1 mixture of ethylacetate and petroleum ether (m.p. 405–407 K).

### Refinement

All hydrogen atoms were positioned geometrically (C–H = 0.93 or 0.96 Å) and were refined using a riding model, with *U*_iso_(H) = 1.2 or 1.5*U*_eq_(C). A rotating group model was applied to the methyl groups.

### Crystal data

**Table:**

C_26_H_20_ClNO_2_	*F*(000) = 864
*M**_r_* = 413.88	*D*_x_ = 1.316 Mg m^−^^3^
Monoclinic, *P*2_1_/*c*	Mo *K*α radiation, λ = 0.71073 Å
Hall symbol: -P 2ybc	Cell parameters from 9882 reflections
*a* = 15.6338 (2) Å	θ = 2.6–30.3°
*b* = 14.0408 (2) Å	µ = 0.21 mm^−^^1^
*c* = 10.0321 (1) Å	*T* = 100 K
β = 108.462 (1)°	Block, colourless
*V* = 2088.82 (5) Å^3^	0.33 × 0.25 × 0.17 mm
*Z* = 4	

### Data collection

**Table:**

Bruker SMART APEXII CCD area-detector diffractometer	6303 independent reflections
Radiation source: fine-focus sealed tube	5132 reflections with *I* > 2σ(*I*)
graphite	*R*_int_ = 0.042
φ and ω scans	θ_max_ = 30.4°, θ_min_ = 2.0°
Absorption correction: multi-scan (*SADABS*; Bruker, 2005)	*h* = −22→22
*T*_min_ = 0.936, *T*_max_ = 0.967	*k* = −19→19
51550 measured reflections	*l* = −14→14

### Refinement

**Table:**

Refinement on *F*^2^	Primary atom site location: structure-invariant direct methods
Least-squares matrix: full	Secondary atom site location: difference Fourier map
*R*[*F*^2^ > 2σ(*F*^2^)] = 0.042	Hydrogen site location: inferred from neighbouring sites
*wR*(*F*^2^) = 0.112	H-atom parameters constrained
*S* = 1.05	*w* = 1/[σ^2^(*F*_o_^2^) + (0.0521*P*)^2^ + 0.7676*P*] where *P* = (*F*_o_^2^ + 2*F*_c_^2^)/3
6303 reflections	(Δ/σ)_max_ = 0.001
273 parameters	Δρ_max_ = 0.38 e Å^−^^3^
0 restraints	Δρ_min_ = −0.40 e Å^−^^3^

### Special details

**Table:**

Experimental. The crystal was placed in the cold stream of an Oxford Cyrosystems Cobra open-flow nitrogen cryostat (Cosier & Glazer, 1986) operating at 100.0 (1) K.
Geometry. All e.s.d.'s (except the e.s.d. in the dihedral angle between two l.s. planes) are estimated using the full covariance matrix. The cell e.s.d.'s are taken into account individually in the estimation of e.s.d.'s in distances, angles and torsion angles; correlations between e.s.d.'s in cell parameters are only used when they are defined by crystal symmetry. An approximate (isotropic) treatment of cell e.s.d.'s is used for estimating e.s.d.'s involving l.s. planes.
Refinement. Refinement of *F*^2^ against ALL reflections. The weighted *R*-factor *wR* and goodness of fit *S* are based on *F*^2^, conventional *R*-factors *R* are based on *F*, with *F* set to zero for negative *F*^2^. The threshold expression of *F*^2^ > σ(*F*^2^) is used only for calculating *R*-factors(gt) *etc*. and is not relevant to the choice of reflections for refinement. *R*-factors based on *F*^2^ are statistically about twice as large as those based on *F*, and *R*- factors based on ALL data will be even larger.

### Fractional atomic coordinates and isotropic or equivalent isotropic displacement parameters (Å^2^)

**Table:**

	*x*	*y*	*z*	*U*_iso_*/*U*_eq_	
Cl1	0.33917 (2)	0.33164 (3)	0.58574 (4)	0.03525 (10)	
O1	0.89366 (6)	0.37834 (7)	0.92588 (10)	0.0275 (2)	
O2	0.99503 (6)	0.88427 (6)	1.10271 (9)	0.02503 (19)	
N1	0.69406 (7)	0.37222 (7)	0.50187 (10)	0.0202 (2)	
C1	0.76533 (8)	0.39716 (8)	0.60758 (12)	0.0194 (2)	
C2	0.61229 (8)	0.36683 (8)	0.52489 (12)	0.0186 (2)	
C3	0.53676 (9)	0.33562 (9)	0.41238 (13)	0.0232 (2)	
H3A	0.5437	0.3220	0.3257	0.028*	
C4	0.45373 (9)	0.32536 (9)	0.42991 (13)	0.0245 (3)	
H4A	0.4046	0.3044	0.3560	0.029*	
C5	0.44385 (8)	0.34703 (10)	0.56185 (13)	0.0230 (2)	
C6	0.51471 (8)	0.37833 (9)	0.67275 (12)	0.0208 (2)	
H6A	0.5063	0.3926	0.7583	0.025*	
C7	0.60098 (8)	0.38886 (8)	0.65627 (11)	0.0175 (2)	
C8	0.67867 (8)	0.41709 (8)	0.76909 (11)	0.0170 (2)	
C9	0.76028 (7)	0.41997 (8)	0.74412 (12)	0.0174 (2)	
C10	0.84595 (8)	0.44340 (9)	0.86210 (12)	0.0195 (2)	
C11	0.87075 (8)	0.54318 (9)	0.89574 (13)	0.0217 (2)	
H11A	0.9200	0.5564	0.9744	0.026*	
C12	0.82630 (8)	0.61647 (9)	0.81913 (13)	0.0204 (2)	
H12A	0.7752	0.6020	0.7441	0.024*	
C13	0.85050 (7)	0.71727 (9)	0.84237 (12)	0.0191 (2)	
C14	0.91311 (8)	0.74920 (9)	0.96894 (12)	0.0196 (2)	
H14A	0.9381	0.7067	1.0420	0.024*	
C15	0.93706 (8)	0.84453 (9)	0.98366 (12)	0.0198 (2)	
C16	0.90136 (8)	0.90856 (9)	0.87281 (13)	0.0226 (2)	
H16A	0.9193	0.9720	0.8824	0.027*	
C17	0.83926 (8)	0.87700 (9)	0.74883 (13)	0.0227 (2)	
H17A	0.8152	0.9195	0.6753	0.027*	
C18	0.81280 (8)	0.78194 (9)	0.73403 (13)	0.0214 (2)	
H18A	0.7699	0.7614	0.6517	0.026*	
C19	0.67020 (7)	0.43834 (9)	0.91020 (11)	0.0177 (2)	
C20	0.70262 (9)	0.37333 (9)	1.01947 (13)	0.0233 (2)	
H20A	0.7325	0.3187	1.0058	0.028*	
C21	0.69045 (9)	0.38998 (10)	1.14923 (13)	0.0269 (3)	
H21A	0.7115	0.3461	1.2217	0.032*	
C22	0.64704 (9)	0.47190 (10)	1.17049 (13)	0.0260 (3)	
H22A	0.6389	0.4829	1.2571	0.031*	
C23	0.61572 (9)	0.53749 (10)	1.06250 (13)	0.0257 (3)	
H23A	0.5872	0.5928	1.0772	0.031*	
C24	0.62681 (8)	0.52078 (9)	0.93238 (12)	0.0221 (2)	
H24A	0.6053	0.5646	0.8600	0.027*	
C25	0.85421 (9)	0.40069 (11)	0.57958 (14)	0.0269 (3)	
H25A	0.8444	0.3918	0.4810	0.040*	
H25B	0.8928	0.3511	0.6316	0.040*	
H25C	0.8822	0.4614	0.6083	0.040*	
C26	1.04176 (9)	0.82146 (10)	1.21483 (13)	0.0257 (3)	
H26A	1.0808	0.8579	1.2908	0.038*	
H26B	1.0769	0.7769	1.1815	0.038*	
H26C	0.9988	0.7875	1.2472	0.038*	

### Atomic displacement parameters (Å^2^)

**Table:**

	*U*^11^	*U*^22^	*U*^33^	*U*^12^	*U*^13^	*U*^23^
Cl1	0.01933 (15)	0.0596 (2)	0.02639 (17)	−0.01311 (14)	0.00657 (12)	−0.01568 (15)
O1	0.0238 (4)	0.0259 (5)	0.0278 (5)	0.0019 (4)	0.0009 (4)	0.0005 (4)
O2	0.0280 (5)	0.0226 (4)	0.0205 (4)	−0.0053 (3)	0.0019 (4)	−0.0048 (3)
N1	0.0225 (5)	0.0224 (5)	0.0164 (4)	−0.0023 (4)	0.0071 (4)	−0.0014 (4)
C1	0.0212 (5)	0.0193 (5)	0.0189 (5)	−0.0015 (4)	0.0079 (4)	0.0005 (4)
C2	0.0210 (5)	0.0197 (5)	0.0143 (5)	−0.0027 (4)	0.0047 (4)	−0.0011 (4)
C3	0.0262 (6)	0.0283 (6)	0.0141 (5)	−0.0031 (5)	0.0050 (4)	−0.0037 (4)
C4	0.0238 (6)	0.0308 (7)	0.0156 (5)	−0.0056 (5)	0.0015 (4)	−0.0043 (5)
C5	0.0179 (5)	0.0307 (7)	0.0192 (5)	−0.0043 (4)	0.0042 (4)	−0.0045 (5)
C6	0.0197 (5)	0.0263 (6)	0.0157 (5)	−0.0039 (4)	0.0046 (4)	−0.0050 (4)
C7	0.0189 (5)	0.0187 (5)	0.0139 (5)	−0.0022 (4)	0.0038 (4)	−0.0014 (4)
C8	0.0190 (5)	0.0170 (5)	0.0140 (5)	−0.0016 (4)	0.0036 (4)	−0.0008 (4)
C9	0.0177 (5)	0.0165 (5)	0.0172 (5)	−0.0019 (4)	0.0045 (4)	−0.0005 (4)
C10	0.0167 (5)	0.0230 (6)	0.0182 (5)	−0.0017 (4)	0.0046 (4)	−0.0018 (4)
C11	0.0166 (5)	0.0241 (6)	0.0220 (6)	−0.0034 (4)	0.0026 (4)	−0.0044 (5)
C12	0.0169 (5)	0.0238 (6)	0.0202 (5)	−0.0043 (4)	0.0054 (4)	−0.0043 (4)
C13	0.0155 (5)	0.0223 (6)	0.0199 (5)	−0.0024 (4)	0.0060 (4)	−0.0034 (4)
C14	0.0181 (5)	0.0221 (6)	0.0183 (5)	−0.0025 (4)	0.0053 (4)	−0.0018 (4)
C15	0.0174 (5)	0.0231 (6)	0.0189 (5)	−0.0030 (4)	0.0057 (4)	−0.0055 (4)
C16	0.0235 (6)	0.0193 (6)	0.0254 (6)	−0.0012 (4)	0.0083 (5)	−0.0026 (5)
C17	0.0213 (5)	0.0241 (6)	0.0224 (6)	0.0009 (4)	0.0065 (5)	0.0004 (5)
C18	0.0173 (5)	0.0256 (6)	0.0199 (5)	−0.0007 (4)	0.0040 (4)	−0.0033 (4)
C19	0.0157 (5)	0.0229 (6)	0.0134 (5)	−0.0045 (4)	0.0029 (4)	−0.0025 (4)
C20	0.0273 (6)	0.0230 (6)	0.0187 (5)	−0.0007 (5)	0.0059 (5)	0.0003 (4)
C21	0.0320 (7)	0.0307 (7)	0.0169 (5)	−0.0034 (5)	0.0062 (5)	0.0030 (5)
C22	0.0281 (6)	0.0350 (7)	0.0160 (5)	−0.0076 (5)	0.0086 (5)	−0.0051 (5)
C23	0.0262 (6)	0.0297 (7)	0.0217 (6)	0.0005 (5)	0.0084 (5)	−0.0056 (5)
C24	0.0226 (5)	0.0257 (6)	0.0168 (5)	0.0007 (4)	0.0042 (4)	−0.0011 (4)
C25	0.0232 (6)	0.0355 (7)	0.0248 (6)	−0.0028 (5)	0.0115 (5)	−0.0004 (5)
C26	0.0249 (6)	0.0303 (7)	0.0192 (6)	−0.0043 (5)	0.0034 (5)	−0.0038 (5)

### Geometric parameters (Å, °)

**Table:**

Cl1—C5	1.7410 (12)	C13—C14	1.4087 (16)
O1—C10	1.2239 (15)	C14—C15	1.3852 (17)
O2—C15	1.3687 (14)	C14—H14A	0.9300
O2—C26	1.4340 (16)	C15—C16	1.4012 (18)
N1—C1	1.3192 (15)	C16—C17	1.3860 (17)
N1—C2	1.3713 (15)	C16—H16A	0.9300
C1—C9	1.4331 (16)	C17—C18	1.3913 (18)
C1—C25	1.5030 (16)	C17—H17A	0.9300
C2—C7	1.4184 (15)	C18—H18A	0.9300
C2—C3	1.4200 (16)	C19—C20	1.3927 (17)
C3—C4	1.3717 (18)	C19—C24	1.3947 (17)
C3—H3A	0.9300	C20—C21	1.3938 (17)
C4—C5	1.4135 (17)	C20—H20A	0.9300
C4—H4A	0.9300	C21—C22	1.386 (2)
C5—C6	1.3703 (16)	C21—H21A	0.9300
C6—C7	1.4176 (16)	C22—C23	1.3876 (19)
C6—H6A	0.9300	C22—H22A	0.9300
C7—C8	1.4291 (15)	C23—C24	1.3901 (17)
C8—C9	1.3765 (15)	C23—H23A	0.9300
C8—C19	1.4931 (15)	C24—H24A	0.9300
C9—C10	1.5163 (16)	C25—H25A	0.9600
C10—C11	1.4643 (17)	C25—H25B	0.9600
C11—C12	1.3395 (17)	C25—H25C	0.9600
C11—H11A	0.9300	C26—H26A	0.9600
C12—C13	1.4644 (17)	C26—H26B	0.9600
C12—H12A	0.9300	C26—H26C	0.9600
C13—C18	1.3956 (17)		
			
C15—O2—C26	117.77 (10)	C13—C14—H14A	120.3
C1—N1—C2	118.32 (10)	O2—C15—C14	124.67 (11)
N1—C1—C9	122.65 (10)	O2—C15—C16	114.72 (11)
N1—C1—C25	117.00 (10)	C14—C15—C16	120.61 (11)
C9—C1—C25	120.35 (11)	C17—C16—C15	119.79 (11)
N1—C2—C7	122.80 (10)	C17—C16—H16A	120.1
N1—C2—C3	117.91 (10)	C15—C16—H16A	120.1
C7—C2—C3	119.27 (11)	C16—C17—C18	120.17 (12)
C4—C3—C2	120.87 (11)	C16—C17—H17A	119.9
C4—C3—H3A	119.6	C18—C17—H17A	119.9
C2—C3—H3A	119.6	C17—C18—C13	120.21 (11)
C3—C4—C5	119.07 (11)	C17—C18—H18A	119.9
C3—C4—H4A	120.5	C13—C18—H18A	119.9
C5—C4—H4A	120.5	C20—C19—C24	119.51 (11)
C6—C5—C4	121.97 (11)	C20—C19—C8	119.73 (11)
C6—C5—Cl1	118.79 (9)	C24—C19—C8	120.72 (10)
C4—C5—Cl1	119.24 (9)	C19—C20—C21	120.13 (12)
C5—C6—C7	119.55 (11)	C19—C20—H20A	119.9
C5—C6—H6A	120.2	C21—C20—H20A	119.9
C7—C6—H6A	120.2	C22—C21—C20	120.08 (12)
C6—C7—C2	119.26 (10)	C22—C21—H21A	120.0
C6—C7—C8	122.54 (10)	C20—C21—H21A	120.0
C2—C7—C8	118.14 (10)	C21—C22—C23	119.98 (11)
C9—C8—C7	117.92 (10)	C21—C22—H22A	120.0
C9—C8—C19	122.16 (10)	C23—C22—H22A	120.0
C7—C8—C19	119.86 (10)	C22—C23—C24	120.20 (12)
C8—C9—C1	120.15 (10)	C22—C23—H23A	119.9
C8—C9—C10	120.32 (10)	C24—C23—H23A	119.9
C1—C9—C10	119.48 (10)	C23—C24—C19	120.10 (12)
O1—C10—C11	121.37 (11)	C23—C24—H24A	120.0
O1—C10—C9	119.18 (11)	C19—C24—H24A	120.0
C11—C10—C9	119.44 (10)	C1—C25—H25A	109.5
C12—C11—C10	123.47 (11)	C1—C25—H25B	109.5
C12—C11—H11A	118.3	H25A—C25—H25B	109.5
C10—C11—H11A	118.3	C1—C25—H25C	109.5
C11—C12—C13	126.17 (11)	H25A—C25—H25C	109.5
C11—C12—H12A	116.9	H25B—C25—H25C	109.5
C13—C12—H12A	116.9	O2—C26—H26A	109.5
C18—C13—C14	119.77 (11)	O2—C26—H26B	109.5
C18—C13—C12	118.73 (11)	H26A—C26—H26B	109.5
C14—C13—C12	121.45 (11)	O2—C26—H26C	109.5
C15—C14—C13	119.40 (11)	H26A—C26—H26C	109.5
C15—C14—H14A	120.3	H26B—C26—H26C	109.5
			
C2—N1—C1—C9	−0.89 (17)	C8—C9—C10—C11	−84.74 (14)
C2—N1—C1—C25	178.93 (11)	C1—C9—C10—C11	97.70 (13)
C1—N1—C2—C7	1.39 (17)	O1—C10—C11—C12	172.29 (12)
C1—N1—C2—C3	−177.10 (11)	C9—C10—C11—C12	−6.60 (18)
N1—C2—C3—C4	177.64 (12)	C10—C11—C12—C13	−176.37 (11)
C7—C2—C3—C4	−0.90 (19)	C11—C12—C13—C18	163.13 (12)
C2—C3—C4—C5	0.5 (2)	C11—C12—C13—C14	−14.47 (18)
C3—C4—C5—C6	0.2 (2)	C18—C13—C14—C15	−0.56 (17)
C3—C4—C5—Cl1	−178.61 (10)	C12—C13—C14—C15	177.02 (11)
C4—C5—C6—C7	−0.5 (2)	C26—O2—C15—C14	7.01 (17)
Cl1—C5—C6—C7	178.35 (10)	C26—O2—C15—C16	−173.30 (11)
C5—C6—C7—C2	0.07 (18)	C13—C14—C15—O2	178.05 (11)
C5—C6—C7—C8	−177.16 (12)	C13—C14—C15—C16	−1.62 (17)
N1—C2—C7—C6	−177.85 (11)	O2—C15—C16—C17	−177.59 (11)
C3—C2—C7—C6	0.61 (17)	C14—C15—C16—C17	2.11 (18)
N1—C2—C7—C8	−0.50 (17)	C15—C16—C17—C18	−0.38 (18)
C3—C2—C7—C8	177.97 (11)	C16—C17—C18—C13	−1.81 (18)
C6—C7—C8—C9	176.38 (11)	C14—C13—C18—C17	2.28 (17)
C2—C7—C8—C9	−0.88 (16)	C12—C13—C18—C17	−175.37 (11)
C6—C7—C8—C19	−1.07 (17)	C9—C8—C19—C20	−71.87 (15)
C2—C7—C8—C19	−178.33 (10)	C7—C8—C19—C20	105.47 (13)
C7—C8—C9—C1	1.36 (16)	C9—C8—C19—C24	110.80 (13)
C19—C8—C9—C1	178.75 (11)	C7—C8—C19—C24	−71.86 (15)
C7—C8—C9—C10	−176.19 (10)	C24—C19—C20—C21	1.01 (18)
C19—C8—C9—C10	1.20 (17)	C8—C19—C20—C21	−176.35 (11)
N1—C1—C9—C8	−0.50 (18)	C19—C20—C21—C22	−0.8 (2)
C25—C1—C9—C8	179.69 (11)	C20—C21—C22—C23	0.0 (2)
N1—C1—C9—C10	177.07 (11)	C21—C22—C23—C24	0.7 (2)
C25—C1—C9—C10	−2.74 (17)	C22—C23—C24—C19	−0.54 (19)
C8—C9—C10—O1	96.34 (14)	C20—C19—C24—C23	−0.33 (18)
C1—C9—C10—O1	−81.22 (15)	C8—C19—C24—C23	177.01 (11)

### Hydrogen-bond geometry (Å, °)

**Table:**

Cg1 and Cg2 are the centroids of the C13–C18 and C19–C24 rings, resepctively.

**Table:**

*D*—H···*A*	*D*—H	H···*A*	*D*···*A*	*D*—H···*A*
C16—H16A···O2^i^	0.93	2.40	3.3005 (15)	163
C18—H18A···Cl1^ii^	0.93	2.78	3.6948 (13)	169
C26—H26B···O1^iii^	0.96	2.54	3.4329 (17)	155
C26—H26C···Cg1^iv^	0.96	2.88	3.8412 (15)	177
C17—H17A···Cg2^v^	0.93	2.97	3.7592 (14)	144

Symmetry codes: (i) −*x*+2, −*y*+2, −*z*+2; (ii) −*x*+1, −*y*+1, −*z*+1; (iii) −*x*+2, −*y*+1, −*z*+2; (iv) *x*, −*y*+1/2, *z*−1/2; (v) *x*, −*y*+1/2, *z*−3/2.
